# Career construction theory: tools, interventions, and future trends

**DOI:** 10.3389/fpsyg.2024.1381233

**Published:** 2024-04-05

**Authors:** Danqi Wang, Yanling Li

**Affiliations:** School of Education, Huainan Normal University, Huainan, China

**Keywords:** career construction theory, My Career Story, career construction interview, career intervention, future trends

## Abstract

With the emergence of the borderless career era in the 21st century, career coaching has experienced a change from career guidance and career education to career counseling. Career construction theory has been widely used in career counseling and has substantial application value. Introducing career construct theory’s assessment tools and intervention strategies is necessary and meaningful. In this mini-review, the qualitative assessment tools and intervention approaches of career construct theory are introduced and analyzed; the qualitative assessment tools include the Career Construction Interview and “My Career Story” workbook, and the intervention approaches include the Computer-Assisted Career Counseling System, workshops, group counseling, and individual counseling. Finally, future research directions are proposed, including an analysis of what kinds of career construction interventions are most effective for which groups and under what conditions, career intervention in the digital age, and the standardization of assessment tools. The novelty of this paper lies in the fact that it purposefully proposes future directions for career construction theory from the perspectives of assessment tools and intervention approaches and that research on the assessment tools and intervention approaches of career construction theory still needs further attention.

## Introduction

Career counseling has changed from career guidance and career education to career counseling. In the 19th century, career counseling was centered on the matching career guidance model, which is making rational decisions based on self and career information. After entering the 20th century, career counseling is based on career development theory, focusing on how individuals make decisions, a process-oriented career intervention. Furthermore, beginning in the 21st century, career counseling centers on career construction theory, focusing on vocational personality, career adaptability, and life theme, emphasizing constructing careers. These three theoretical models are the career guidance model to determine the person-job match, career education to promote career development, and career counseling to design work-life.

### Career construction theory

Savickas (2005) proposed the career construction theory based on personal constructivism, social constructionism, and post-modernity. Career construction theory believes that the essence of individual career development is the dynamic construction process of pursuing mutual adaptation between the subjective self and the external objective world, and different people construct different stories. Career construction theory provides a dynamic perspective to give personal meaning to memories, present experiences, and plans, constructing careers through a sense of meaning and clarifying future directions. The theory includes three parts: vocational personality, career adaptability, and life theme. Occupational personality refers to an individual’s career-related abilities, needs, values, and interests. Career adaptability is described as “a psychosocial construct that denotes an individual’s resources for coping with current and imminent vocational development tasks, transitions, traumas” (Savickas and Porfeli, 2012, p. 662). The difference between occupational personality and career adaptability is that occupational personality emphasizes the content of a career, while career adaptability emphasizes the coping process of constructing a career. Career adaptability deals with how individuals construct careers, while occupational personality deals with what careers they construct. Career adaptability deals with how individuals construct their careers, while occupational personality deals with what careers they construct. Unlike vocational personality and career adaptability, life theme is a dynamical system that primarily explains why individuals make career choices and the significance of those choices and expresses the uniqueness of the individual in a particular context, which provides a way of looking at the world. Career counseling, developed from career construction theory, focuses on vocational personality, career adaptability, and constructing meaning in life themes (Savickas, 2013). Vocational personality focuses on the “what,” career adaptability is about the “how,” and life theme responds to the “why” (Guan and Li, 2015).

Compared to other career theories, career construction theory helps students adapt to the future’s complex and changing career world and inspires a richer perspective on career development (Gao and Qiao, 2022). Meta-analysis has shown that social construction theory is more effective than individual-environmental matching theory (Langher et al., 2018). Career construction theory seeks to explain the interpersonal process in which individuals construct the self, establish the direction of career behavior, and assign meaning to careers, providing a unique perspective on how to view the subject of career counseling (Hou et al., 2014). Career construction theory provides specific ideas to help the case make career decisions and enhance work satisfaction (Savickas, 2005). Therefore, this review aims to introduce the tools, interventions, and future directions of career construction theory to help individuals better adapt to the rapidly evolving situation.

To ensure the quality of the literature, the terms “career construction” and “intervention” were used as search terms in this study, both of which appeared in the title, abstract, or keywords. A comprehensive search was conducted on the “Web of Science, PsycINFO, and EBSCO.” The search was limited to English-language articles. Specifically, the literature was searched from 2013 to 2023. In addition, only standard research papers were included in this study, excluding review-type articles.

### Life design counseling

Life design counseling is based on career construct theory, which gives meaning to life and supports adaptive responses by helping the individual to tell a career story, constructing the past, present, and future to form continuity and consistency. The five assumptions of the life design model of vocational intervention are contextual possibilities, dynamic processes, non-linear progression, multiple perspectives, and personal patterns. Life design counseling is lifelong, holistic, contextual, and preventive. It aims to increase the client’s adaptability, narratability, and activism (Savickas et al., 2009).

The life design paradigm relies on story construction and action. The first stage of life design counseling is constructive, which involves clarifying the problem and what one hopes to achieve through counseling. The counselor encourages the client to find the life theme by describing the problem to be solved through a story. The second is deconstruction, which helps the client reflect on and shape the story by allowing them to clearly express experiences, expectations, actions, and expectations for the future. The third stage is reconstruction. The counselor and the client can interpret the story from different perspectives, thus enabling the client to rewrite his or her story. The fourth is the co-construction stage. The issues raised by the client are put into the rewritten story, and a new story is co-constructed as a solution. The fifth stage is action. Assign participation in some of the narrative’s possible self-relevant activities. It is necessary to specify what they will do and what this means to help the client make a plan (Savickas et al., 2009).

### Career construction interview

The Career Construction Interview is a structured process based on life design counseling designed to help clients tell, hear, and enact their life career stories. Counselors help them to coherently tell their career story, cope with changes in the environment, design a meaningful life, and take action by conducting a qualitative career assessment with a narrative model and methodology. The career construction interview comprises five questions, each leading to a thematic story. Role Models are to identify adjectives that describe self-constructs and concepts. Favorite magazines/TV/websites are to identify the types of environments and activities that interest the client. Favorite stories are understanding the stories or cultural scripts the client might use to envision transformational outcomes. Favorite mottos can give the client some advice. Early recollections can provide insight into how the client perceives the issues presented in the transition narrative (Savickas, 2011).

At the beginning of the second phase, the counselor draws a portrait of life-occupation based on the client’s answers to CCI questions, combined with observation and reflection. By reviewing the story together and encouraging reflection and reflexivity in the conversation, the counselor and client construct a powerful new life-career identity that has coherent meaning for the client’s life. In the third phase, the client develops an action plan with the counselor. The career interest results obtained from the CCI correlate moderately with the quantitative Career Interest Inventory results, which suggests that the CCI agrees with traditional quantitative assessment tools (Barclay and Wolff, 2012). Barclay (2018) provided three additional ways to use the CCI: written exercises, career collages, and career portfolios. Lindo and Ceballos (2019) developed the Child and Adolescent Career Construction, which includes the development of appropriate expressive arts to promote self-expression and career exploration in children ages nine and older. The CACCI includes a socio-emotional focus that encourages clients to explore self-concepts, life themes, and career awareness.

### My Career Story

“My Career Story” is a career autobiographical workbook developed based on the Life Design Paradigm and contains written exercises and goal-setting activities essential to successful career planning (Brown and Ryan Krane, 2000). It corresponds to the construction, deconstruction, reconstruction, co-construction, and action of life design counseling (Hartung and Santilli, 2017).

MCS is designed to help clients tell, hear, and enact their life-career stories about who they are, who they want to be in the world of work, and how they can connect themselves to careers they might enjoy. Individuals, groups, and educators can use MCS to guide self-reflection to increase narrative identity, intentionality, and adaptability in career planning, career choice, and work adjustment. The MCS workbook consists of three sections to guide clients in telling their life stories. The first section, “Telling My Story,” begins by defining the participant’s problem, listing the careers they have considered pursuing and how they would like the workbook to benefit them. Next, participants answered questions related to life-career topics: (1) role models they admired while growing up, (2) favorite magazines and television shows, (3) stories from favorite books or movies, (4) favorite mottos. The second part is “Listening to My Story.” Portrait their lives by integrating smaller stories into a more cohesive career story. Including (1) Who will I be? (2) Where do I like to be? (3) The portrait summarizes (4) Rewrite my story. The third section, “Enacting My Story,” involves creating a realistic plan to implement the story (Santilli et al., 2019). The MCS can be used by clients alone or with the counselor’s assistance. As an adjunct to career counseling, the MCS can be used in one-on-one individual and group counseling and career development learning activities in the classroom or other settings (Hartung and Santilli, 2017).

### Career intervention

**Figure 1:**
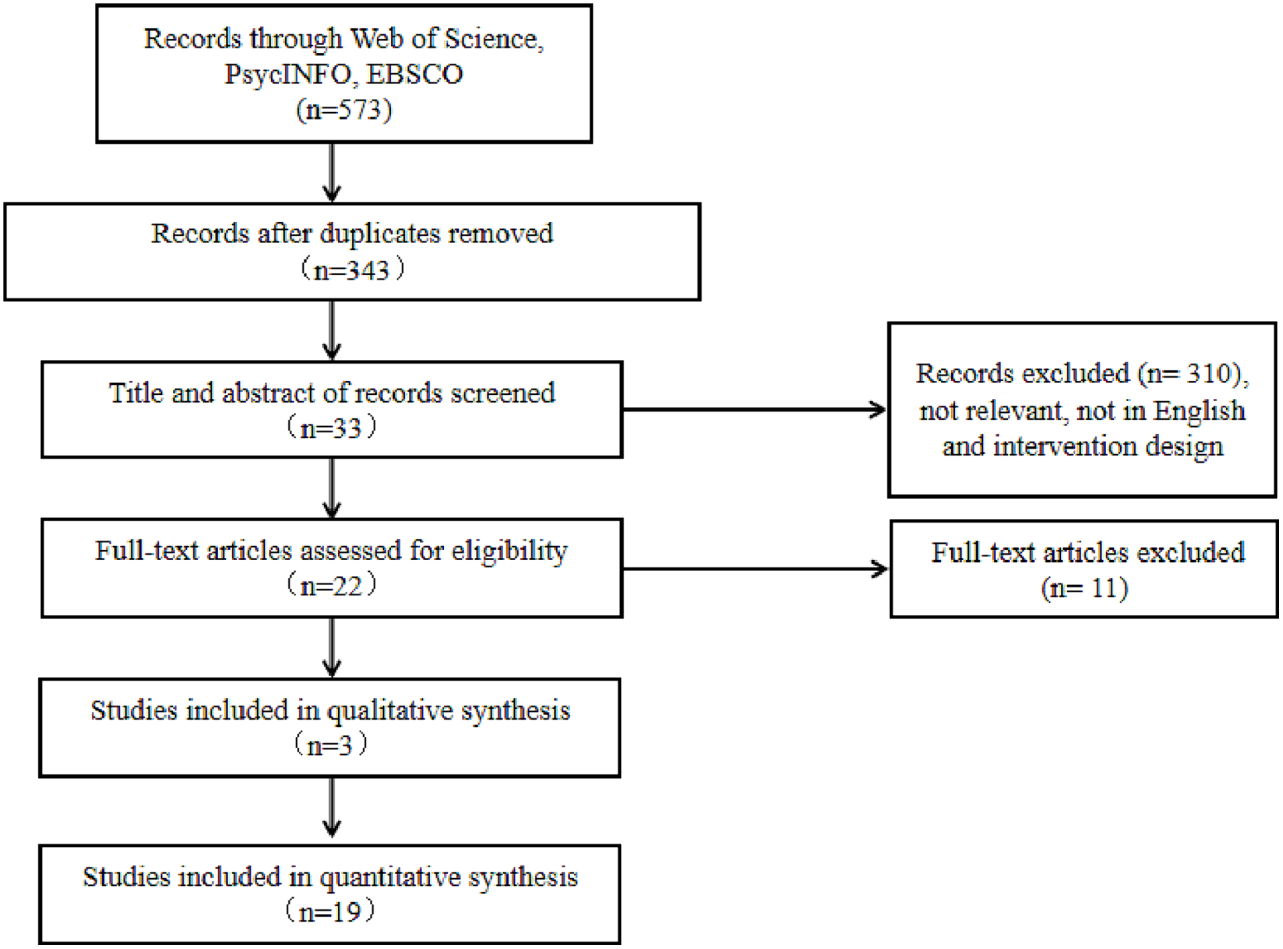
PRISMA flow chart.

Twenty-two studies published between 2013 and 2023 met all criteria and provided the necessary data for the systematic review. Databases included Web of Science, PsycINFO, and EBSCO. Two authors screened all titles and abstracts. In addition, they considered the eligibility of full-text articles. First, the databases were searched with the keywords “career construct theory” and “intervention.” Furthermore, a citation search was conducted for key papers, and reference checking was performed as suggested by Tuttle et al. (2009). Thus, the search strategy was iterative and multi-stage, including computerized and manual searches. Therefore, it can be concluded that these searches were adequate for a systematic review. Finally, 22 studies were identified, including three qualitative and 19 quantitative studies. The two authors evaluated these studies against the selection criteria and agreed on the final 22 studies. Figure 1 depicts the process of selection.

These studies review intervention research on career construction theory, as shown in Table 1.

**Table 1:** Intervention studies.

Authors/date	Method	Quantitative data	Strategies
1.Cardoso et al. (2022)	Group	Vocational identity, Career adaptability	Life Design counseling
2.Maree (2019)	Group	Career adaptability	Life Design counseling:Career Construction Interview
3.Maree et al. (2019)	Group	Career adaptability	Life Design counseling:Career Construction Interview
4.Gai et al. (2022)	group	Career adaptability	Motivational interview: engaging, focusing, evoking, and planning
5.Cook and Maree (2016)	Group	Career adaptability	Life Design counseling:Career Construction Interview
6.Maree et al. (2017)	Group	Career adaptability	Life Design counseling:Career Construction Interview
7.Obi (2015)	Seminar	Indecision, Anxiety, Uncertainty	Life Design counseling
8.Maree and Symington (2015)	Seminar	Career adaptability	Life design counseling:Career Construction Interview
9.Cadaret and Hartung (2021)	Seminar	Vocational identity, Career adaptability	“My Career Story” workbook
10.Ginevra et al. (2017)	Seminar	Career adaptability	“My Career Story” workbook
11.Peila-Shuster et al. (2021)	Seminar	Career adaptability	“My Career Story” workbook
12.Da Silva et al. (2022)	Seminar	Career adaptability, Vocational identity, Student career construction inventory	Career Construction Interview
13.Santilli et al. (2019)	Seminar	career adaptability, hope, optimism, resilience, future orientation	“My Career Story” workbook
14.Cardoso et al. (2018)	Seminar	Vocational certainty, Career maturity, Career decision self-Efficacy	“My Career Story” workbook
15.Nota et al. (2016)	Online	Career adaptability, Life satisfaction	Life design counseling
16.Pordelan et al. (2018)	Online	Career development	Life design counseling:Career Construction Interview
17.Pordelan et al. (2021)	Online	Career decision, Career decision-making self-efficacy	Life design counseling:Career Construction Interview
18.Zammitti et al. (2023)	Online	Resilience, Subjective risk intelligence, Career adaptability, Self-efficacy, Optimism, Hope, Life satisfaction	Life design counseling
19.Camussi et al. (2023)	Online	Career adaptability, Courage, Time perspective, Resilience	Life design counseling
20.Maree and Twigge (2016)	Individual		Career Construction Interview
21.Maree and Gerryts (2014)	Individual		Career Construction Interview
22.Maree (2016)	Individual		Career Construction Interview

### Career group

Career Group is based on group counseling theory and can promote the cognitive, emotional, and behavioral aspects of an individual’s career development. Career group guidance and career group counseling are two forms of career groups. The difference is that career group guidance has more participants and focuses on transferring knowledge. Career counseling has fewer members and emphasizes interaction and communication between members (Jin, 2007).

Researchers examined the effects of life design group guidance on 9th grade. Findings supported the effects of life design group guidance on career identity, career adaptability, and career decision-making self-efficacy (Cardoso et al., 2022). Maree (2019) used quantitative and qualitative research methods to conduct group career construct counseling with 11th-grade students. The Career Adaptability Scale was used for quantitative analysis. Career interest analysis and Maree Career Matrix were used for qualitative intervention. The findings revealed that students significantly improved career adaptability. Maree et al. (2019) explored the impact of life design group counseling on unemployed young adults’ career adaptability. First, the Career Interest Profile was used to obtain information about career choices: work-related information, five most and least preferred career preferences, six career choice questions, and 15 career story narrative questions. Career counseling techniques such as career genealogy charts, interviews, and personal statements were used. Results indicated that life design group counseling increased participants’ career adaptability.

Recently, Gai et al. (2022) used career construct theory to develop a peer motivational interview that included engagement, focus, arousal, and planning. The research involved senior students conducting one-on-one career motivational interviews with junior students. Results indicated that the intervention increased students’ career control and career confidence. Cook and Maree (2016) compared the effects of career construction group counseling and a life-oriented curriculum on 11th-grade students in different educational settings. The group counseling included Collage, the Career Interest Profile, and the lifeline technique. Participants demonstrated higher career adaptability after participating in career construction group counseling. Maree et al. (2017) used career construction group counseling. The experimental group completed narrative questions in the Career Interest Profile. They created career collages depicting how they see their future. In addition, “My Lifeline” was drawn to mark essential themes in their lives. The quantitative study results indicated that life design group counseling did not increase participants’ career adaptability compared to the traditional program.

### Seminar

Seminar is another form of group counseling. Seminars are less frequent and intensive than group counseling, with more fixed topics and less interaction between members, making them an efficient method (Jin, 2007).

Life design counseling can reduce indecision, anxiety, uncertainty, and insecurity among college students (Obi, 2015). Maree and Symington (2015) designed eight life design workshops with five 11th-grade students in a private school. The students demonstrated increased effort to address issues related to career concern, control, curiosity, and confidence, suggesting that the intervention facilitated the development of their career adaptability. Cadaret and Hartung (2021) designed career construction group counseling using the workshop format combining individual reflection and group discussion. The workshops were based on the My Career Story (MCS) workbook. The first session was “Telling My Story,” which included role models, favorite magazines/TV shows/websites, favorite books/movies, and favorite mottos. The second was “Hearing My Story,” which included describing myself and my interests, scripting roles, making suggestions, and constructing a life portrait. The third session, “Enacting My Story,” included co-setting goals, seeking more information, and exploring pathways to select and identify career goals. Results indicated that the career construction intervention increased students’ career control and confidence. Ginevra et al. (2017) used a life design approach to develop resources that help cope with career transitions, encourage thinking about the future, identify one’s strengths, and plan future projects. It is divided into three phases. Participants were encouraged to tell, revise, and construct their career stories in the first phase. In the second phase, participants were administered an online questionnaire on hope, optimism, resilience, future direction, and career readiness. Consider their strengths in response to the career change in the third phase. Results indicated that the life design approach improved their career adaptability.

Peila-Shuster et al. (2021) used career construct theory to conduct career workshops with adults who had been unemployed for more than six months. Workshops included current status, describing role models, favorite mottos, rewriting stories, reflections, and action plans. The counseling utilizes the My Career Storybook to help participants cope with their problems and prepare for their job search by facilitating narratability, intentionality, and career adaptability. Da Silva et al. (2022) conducted career construct interviews with students. The interview consisted of three workshops that (1) discussed role models, television shows, books or movies, mottos, and early memories; (2) Exploring participants’ answers to career construct interview questions; (3) Discuss the steps needed to implement a new career plan. The study showed that the Career Construct Interview promotes the development of students’ career adaptability and remains stable 3 months after the intervention. This suggests that the intervention of the Career Construct Interview has a good latency. Santilli et al. (2019) compared the impact of life design and traditional career counseling on adolescents. Life design group counseling utilized the “My Career Story” workshop format. The results showed that the intervention promoted the development of career adaptability in the Life Design group. This suggests that “My Career Story” may be an effective means of developing career adaptability in adolescents.

However, the study yielded inconsistent results. Researchers examined the impact of the life design workshop on 9th and 12th-grade students. The intervention utilized the “My Career Story” life design methodology. The results showed that the life design intervention did not impact students’ career adaptability (Cardoso et al., 2018).

### Online career group

The advantage of online interventions is the availability of audiovisual materials, including videos, slideshows, and animations, which help students explore values, interests, and skills independently. Online career counseling is more accessible than traditional career guidance, and students can access various practical information (Chen et al., 2022).

Nota et al. (2016) compared the validity of online life-based design and traditional test interpretations. All students received personalized feedback, including suggestions for future schools and jobs related to their interests, values, and motivation. Results indicated that the online life design group demonstrated higher career adaptability, life satisfaction, and future aspirations. The researchers compared online and face-to-face life design counseling on career development. The online interventions included an introduction to online books, bilingual career videos, short animations, access to a virtual library, an introduction to similar websites that promote career development, and online chats with career counselors. The results showed that online and face-to-face career interventions improved students’ career development (Pordelan et al., 2018). Later, they compared life design digital storytelling and face-to-face storytelling, and the study found that the digital storytelling group had higher career decision self-efficacy than face-to-face storytelling (Pordelan et al., 2021).

Zammitti et al. (2023) conducted a life design paradigm online career intervention with college students to enhance their psychological resources. The online intervention consisted of career workshops and 13 online activities. The study showed that an online group career intervention in the life design paradigm promotes the development of resilience, subjective risk intelligence, career adaptability, self-efficacy, optimism, hope, and life satisfaction. Camussi et al. (2023) foster the development of student’s skills to face complexity and unpredictability, transforming their time perspective into optimism to face the future. The intervention was based on the theoretical model of Life Design. The intervention themes were “My future, why?” and “Who am I and who do I want to be?.” The intervention consisted of two online workshops. It included reflections on conscious life design and current global contextual challenges. The study demonstrated that the Life Design online intervention facilitated the development of students’ levels of career adaptability, courage, time perspective, and resilience.

### Individual career counseling

Individual career counseling is usually a one-on-one approach that assists with career confusion to enhance career adaptability. Individual counseling has the highest cost but significantly impacts the client and the counselor.

The value of individual career counseling is to help all those challenged by unemployment and poverty (especially emerging adults) to become employable, find decent work, and increase their sense of self, and in the process, promote the idea of a fair and just society (Maree and Twigge, 2016). Maree and Gerryts (2014) conducted narrative counseling with a newly young male engineer based on career construct theory. Methods included collage, Career Interest Profile, life chapters, lifeline, early recollections technique, and Career Construction Interview. Participants demonstrated an increase in willingness to cope with challenges and adaptive strategies. This suggests that narrative counseling can facilitate the development of career adaptability. Maree (2016) conducted career construction counseling with a mid-career Black man to construct, deconstruct, reconstruct, and co-construct the client’s life story. The interview included role models, favorite magazines/TV/websites, favorite stories, early memories, and favorite mottos. The client demonstrated an improved self-awareness and a willingness to be more flexible in dealing with challenges related to the career.

## Discussion

### Career assessment

The Career Construction Interview and MCS workbook are two qualitative assessment tools under the Career Construction Theory. The groups for which the tools are applicable may be different. The adult population may be more suitable for the Career Construction Interview, and most individual counseling uses the Career Construction Interview (Maree and Gerryts, 2014; Maree, 2016; Maree and Twigge, 2016). Quantitative tools for career construction intervention mostly use a career adaptability scale (Maree and Symington, 2015; Ginevra et al., 2017; Maree et al., 2019).

Quantitative assessment is a standardized and scientific measurement tool but has certain disadvantages. The advantage of qualitative assessment is that it facilitates the discussion of group career counseling and can improve the shortcomings of quantitative tools. The case study of self-narrative can help the researcher to sort out the main conflicts and critical variables in career development (Guan and Li, 2015). Integrative Structured Interview, based on the system’s theoretical framework, is a method that combines qualitative and quantitative measures to advance storytelling. Using Hollander’s interests as the basis for quantitative assessment, integrating assessment results with storytelling, the integrative structured interview facilitates this integration through quantitative score-based career storytelling that focuses not on the scores but on facilitating participants’ understanding of their scores, career decisions, and transitions (McMahon et al., 2020). Therefore, it is necessary to develop a hybrid standardized assessment method based on career construction theory.

### Career intervention

Career construction theory has been widely used in the field of career counseling. Career group counseling is guided by the theory of career construction, and career intervention programs are designed for the career construction process of different groups, which can effectively solve the problems faced by different groups in career development. Individual career counseling can help cases to link their self-concept with their work through the self-construction of work so that individuals can become the creators of their work and actively construct the meaning of their careers to be prepared for the new changes in the work pattern.

Brown and Ryan Krane (2000) meta-analysis identified five critical elements of career counseling: written exercises and workbooks, individualized explanations and feedback, career world information, role modeling, and building support. The MCS workbook corresponds to the written exercises and workbooks among the key elements. Another meta-analysis indicated that the three critical elements of career counseling are counselor support, value clarification, and psycho-educational intervention (Whiston et al., 2017). The career construction interview gained direct counselor support and clarification of specific values. Combining the MCS manual and supporting materials may effectively develop career adaptability in adolescents (Santilli et al., 2019). However, some research suggests that ninth graders show more difficulty than twelfth graders in recounting their own experiences (Cardoso et al., 2018). This may be because the career construction interview is more helpful for lower grades, which require direct support and clarification of specific values from the counselor.

Currently, the primary interventions of career construction theory are computer-assisted career counseling systems, workshops, group counseling, and individual counseling. Career courses are the most effective (Oliver and Spokane, 1988). Therefore, converting life design counseling into a career course is warranted, and a career construction orientation curriculum needs to be developed to enrich the career construction intervention. A meta-analysis by Whiston et al. (2003) demonstrated that intervention effectiveness significantly increases using a computerized career guidance system in counseling. Various career intervention approaches are often integrated into practice, mainly using computerized career guidance with other modalities. The study found that a comprehensive intervention combining online life design and written exercises was more likely to increase students’ career adaptability and life satisfaction (Nota et al., 2016).

## Future research trends

### Career assessment

Although the assessment tools for career construct interventions have been enriched in recent years, the stability, validity, and applicability of the assessment tools still need to be tested in the further. Career construction interventions focus on the reconstruction of life stories. Some studies have found that career construct interventions did not increase students’ career adaptability (Maree et al., 2017; Cardoso et al., 2018). This suggests that relying on the Career Adaptability Scale as a quantitative study is insufficient, some questionnaires should be designed to measure whether students can articulate and identify what is important to them before and after the intervention. Assessment tools for career construction intervention mainly consist of qualitative or quantitative tool, but standardization still needs to be improved (Di Fabio, 2016; Cardoso et al., 2022). Some studies utilize quantitative and qualitative assessment tools (Maree, 2020), but they need more cross-cultural validation.

Therefore, future research in assessment tools can consider the following aspects: In terms of assessment content, special assessment tools must be prepared for different and unique groups. Savickas (2011) developed a complete set of guidelines for career construction counseling. Online guidelines and assessment tools could be developed in the future, incorporating technologies such as computer networks and multimedia. In particular, comprehensive assessment tools that include quantitative and qualitative aspects should be developed to meet the needs of large-scale research with different groups and achieve standardization and stability of assessment methods.

### Career intervention

First, there is a question of what groups and career interventions are most effective under what conditions. The economic benefits of career interventions in different modalities, age groups, and various intervention goals are critical. The meta-analysis result indicated that the career course was the most effective but required the most intervention time. Individual counseling produced more benefits per session than other interventions (Oliver and Spokane, 1988). Subsequently, meta-analysis yielded different results. Individual career counseling was the most effective, followed by group career counseling, with career courses coming in third. Computerized online systems were the most cost-effective (Whiston et al., 1998). A recent meta-analysis indicated that individual counseling was the most effective, while group and individual counseling and computer-based interventions varied widely (Whiston et al., 2017). Meta-analyses have not yet yielded consistent conclusions. In addition to the results, individual and group counseling are effective methods. However, at the same time, it is essential to consider the number of people and the economic benefits that professional interventions can bring (Whiston, 2011).

Additionally, the results showed differences in intervention impact based on the participants’ grades. Ninth graders only improved at the level of career certainty, while twelfth graders showed more significant development on all measured variables. This may be because higher-graders can better understand what is important to them and what they strive for. Therefore, it is essential to consider the characteristics and needs of different groups to maximize the effectiveness of career construction interventions in future research. Different intervention modalities affect individuals’ career development, which is best for group counseling and which works best for individuals. These issues must be better understood, requiring meta-analysis or systematic review to explore in the further.

Second, digital technology is essential for career interventions. In particular, Online interventions allow alternative experiences and role modeling to be more readily available through websites where short videos of successful people can be viewed and inspired. Therefore, career construction theory may benefit career interventions in the digital age. Online career construction interventions are very efficient and likely to be used more and more. Online career construction can present stories in short films, slideshows, or photographs, allowing the client and the counselor to discover hidden stories and help the client gain new concepts. The advantage of online career construction intervention is convenience, where stories can be opened on a computer or other electronic device. In the storytelling process, information technology is utilized as a platform for digital storytelling, where one’s life story is expressed as a photo, movie, or audio (Pordelan et al., 2021). In the future, personalized interpretation and feedback procedures can be added to the computer-based online intervention to maximize the usefulness of the career construction intervention.

Finally, developing new content and a short career construction interview are necessary. Using career construction theory, the researcher developed a peer motivational interview for at-risk students that included engaging, focusing, evoking, and planning (Gai et al., 2022). Questions include “What do you want to obtain from your future occupation? Why?” “What occupations are you likely to pursue in the future? What occupations are you unlikely to pursue? What occupations are you not sure about whether to pursue? How can you become certain?” Future research needs to focus on particular groups as subjects, focusing on those severely hindered in their career development or career transition, and test the effectiveness of career interviews through group interventions to maximize the effects of career interventions.

However, completing the career construction interview typically requires two 90-min sessions, which hinders its practical use with many students in school. Therefore, Rehfuss and Sickinger (2015) developed a short form of the career construction interview. Only three initial career construction interview questions were used in the short form. “Who did you admire when you were growing up? What are your favorite magazines, TV shows, or websites? Tell me your favorite saying or motto.” These three questions were used to learn about the students’ role models, self-advice to help solve current problems, and preferences for the work environment. In addition, there is a need to develop a short form of the Life Design Group Counseling and MCS. Also, some form of screening is necessary to determine what questions of the career construction intervention will benefit the individual the most.

## Conclusion

Career construction theory applies to the current borderless career era, and such a career theory perspective is more helpful for individuals to adapt to the complex and changing career world in the future. Currently, the tools of career construction theory mainly include the structured career construction interview and the qualitative assessment manual of MCS. The interventions of the theory mainly include workshops, group counseling, online group counseling, and individual interviews. This study identified several challenges to the career construction tools and interventions.

Therefore, it offers some suggestions on how to deal with these challenges: Future researchers need to pay attention to the development of comprehensive quantitative and qualitative assessments to standardize and stabilize assessment methods for the tools. For the interventions, there is a need to examine the question of what groups and under what conditions career interventions are most effective. Second, future research should develop personalized interpretation and feedback procedures for computerized online interventions in the digital age. Finally, developing new content and a short career construction interview are necessary.

## Author contributions

DW: Writing – review & editing, Writing – original draft. YL: Writing – review & editing.
